# Expert Evaluation of Deep Learning Approaches to White Matter Hyperintensity Segmentation in Older Adults

**DOI:** 10.1002/alz70856_106019

**Published:** 2026-01-11

**Authors:** Adam Martersteck, Siobhan McDermott, Caleb VanDyke, Karthik Sreenivasan, Maria Kharitonova, Sophia Moore, Rhiana Schafer, Amanda Cook Maher, Elizabeth Finger, Felicia C. Goldstein, Ozioma C. Okonkwo, Angela C. Roberts, Emily J Rogalski, SuperAging Research Initiative

**Affiliations:** ^1^ Healthy Aging & Alzheimer's Research Care (HAARC) Center, University of Chicago, Chicago, IL, USA; ^2^ University of Chicago, Chicago, IL, USA; ^3^ University of Michigan, Ann Arbor, MI, USA; ^4^ Western University, London, ON, Canada; ^5^ Emory University School of Medicine, Atlanta, GA, USA; ^6^ University of Wisconsin‐Madison School of Medicine and Public Health, Madison, WI, USA

## Abstract

**Background:**

White matter hyperintensities (WMHs) are critical markers of cerebrovascular health and neurodegenerative disease. Accurate and reproducible quantification of WMHs is essential for characterizing vascular contributions to aging, cognition, and Alzheimer disease and related dementias. Deep learning pipelines have emerged as powerful tools for WMH segmentation, yet limited research compares their performance using expert evaluation as the benchmark. Here, we assess the performance of five deep learning WMH segmentation pipelines by comparing their outputs through blinded neuroradiologist ratings.

**Method:**

We processed FLAIR scans from 100 older adults (aged 80 and older) enrolled in the SuperAging Research Initiative. 3D T_2_‐weighted FLAIR and T_1_‐weighted MPRAGE sequences followed the ADNI‐3 protocol, acquired across five sites, using 3T scanners from three vendors (GE, Siemens, Philips). Binary segmentation masks from five deep learning pipelines were utilized: sysu_media, ANTSx, DeepWMH, TrUE‐Net, and HyperMapp3r. A neuroradiologist (C.V.) evaluated the per‐participant level randomized segmentation masks, overlaid on the FLAIR and T_1_‐weighted image, using a 7‐point Likert‐type scale, where 1 indicated “poor segmentation” and 7 indicated “excellent segmentation". Ratings were based on anatomical plausibility and alignment with WMH voxels visible on FLAIR. To compare scores, a Kruskal‐Wallis test and post‐hoc Mann‐Whitney pairwise comparisons were used.

**Result:**

The Kruskal‐Wallis test revealed significant differences in segmentation quality across the five pipelines (*p* = 7.73 x 10^‐43^). Post‐hoc Mann‐Whitney tests showed ANTSx (mean rating = 5.59 ± 1.17) performed significantly better than all other pipelines (all *p* < 0.00001), while HyperMapp3r (mean rating = 2.33 ± 1.22) consistently received significantly lower ratings (all *p* < 0.00001). DeepWMH (mean rating = 4.45 ± 1.34), sysu_media (mean rating = 4.18 ± 1.20), and TrUE‐Net (mean rating = 4.49 ±1.18) had comparable ratings, with no significant differences between the three.

**Conclusion:**

This study highlights significant variability in the quality of WMH segmentation across commonly used deep learning pipelines when benchmarked against expert evaluation. Among the evaluated pipelines, ANTSx demonstrated superior performance, producing clinically plausible segmentations with high anatomical fidelity. These findings underscore the importance of expert validation in selecting and refining automated segmentation tools for research and clinical applications in aging and neurodegenerative disease.

